# Discussions at the Seventh Annual Meeting of the Ohio State Dental Society

**Published:** 1873-02

**Authors:** 


### Discussions at the Seventh Annual Meeting of the Ohio State Dental Society.

### THE BURRING ENGINE.

Dr. W. M. Herriott observed that the use of the burr for excavating teeth dated back a great while. A variety of forms had been given to handles used in connection with the burr-drill but it was only recently that a machine had been developed to run the burr and make it practical for dental uses. He gave preference to Morrison's engine, because it was noiseless and easily controlled.

Dr. B. F. Rosson had had no experience with any burring engine except Morrison's, and of course could not speak for or against any others. He had no doubt many others were valuable. His experience with Morrison's had been very favorable. He had been enabled to accomplish results with this machine that were satisfactory to himself. It is certainly a great labor-saving machine. He concurred in the remarks of the gentleman in reference to the temptation to sacrifice a portion of the natural teeth. He believed he had been guilty of cutting away more from the teeth than was actually necessary in some cases, because it was so much more easily done than by the old manner that one could hardly stop when enough was cut away. He was however, very favorably impressed with the Morrison machine. A portion of a machine would be exhibited to them by Mr. Arbaugh, of Piqua, having water for its motive power, with which he thought they would be favorably impressed, when they examined it. In regard to the Morrison engine he had but one objection—he was going to say two: one being that of having to move it out of the way sometimes, but that was so trifling that he believed he only had one serious objection to it; that was that it did not want to work in his absence.

Dr. D. R. Jennings said he supposed all were aware that they could not cut enamel, if an attempt is made to cut or file it it almost always breaks. The only way is to grind it down with the burr if you wish to cut it down. The burr is nothing more nor less than a set of little chisels which chip it off, under a glass this can readily be observed. It is from the form of the burr and the manner of applying it that it accomplishes the work. After cutting down to the enamel with a chisel, by then inserting the point of this burr you can run it right under the enamel, and chip it off very nicely. There is no trouble about cutting the teeth down too much. He thought the Morrison engine one of the best, but thought all would like the electric engine when they saw it in use.

The pneumatic was less difficult to move around, and furnished all the force necessary. He did not see why some wanted to apply so much force; force or power was not what was so much needed; it was the adaptability, and to know how to use them properly. If the operator bears on quite hard it may heat the tooth much more rapidly but may not cut much faster. A man with a file does not cut so much by the force he puts on as by the manner in which he uses it. So with the burring engine.

Dr. L. Buffett had little experience with the burring engine and could not advocate one more than another. Each dentist should use the instrument that suited him best. As a labor-saver the burring-engine is of great service; he could make an excavation of a given size with it, in perhaps one-third the time it would take to accomplish it in the old way. In large operations in trimming large crown fillings he formerly found himself pretty well tired out, and unable sometimes to finish the job at one sitting. Now it was really easy to trim down the fillings and complete the work. So for as the objection is made to the burring engine tearing the gold, he would say if it tore the gold it ought to be torn.

Dr. D. R. Jennings did not wish to be misunderstood with regard to the use of the burring engine. He held that in excavating with burrs the excavation is smoother, the walls nicer, the cavity in better shape, cleaned out nicer, and the teeth less liable to decay. It was no detriment to the teeth to be thus thoroughly cleaned out, and was better for the patient. The sacrifice of the teeth in such a case was no sacrifice but a real benefit.

Dr. W. M. Herriott said, lest some might suppose he was trying to advertise a particular kind of machine, he simply spoke of the Morrison machine as one that seemed to suit him the best. As Dr. Buffett had said no two men have exactly the same ideas concerning such things, and they must reach the same object in different ways, by using instruments somewhat different. There are of course objections to the Morrison machine. There is a machine now being gotten up by a Mr. Green, not yet perfected, that is valuable on account of being easily controlled. The pneumatic engine has its advantages in being easily placed in different directions, and used in ways that the Morrison engine cannot be used. In regard to cutting the teeth away too much, he referred to the discussion of this matter at the meeting of the American Dental Association last summer at Niagara, where two prominent members differed; one advocating the plan of cutting away on the outside so that the gold would show, and the other advocating the idea of cutting away on the inside, thus saving the external form of the tooth, and showing that rather than the gold. He believed with Dr. Jennings that it was best to cut away, cleaning the teeth out thoroughly and perfectly.

In using the burr in cutting down fillings there is danger of breaking the enamel, and for that reason great care should be exercised, and the burr not be allowed to come in contact with the fillings, and the very small points only should be used in connection with the engine.

Dr. Siddall said he had been using a burring engine for a number of months, and liked it very much. He found one difficulty in using it which he desired to know how to obviate, that was where it was run very rapidly, as it sometimes must be, there is a great deal of heat produced, so much so that he had to withdraw it frequently or use it very slowly.

Dr. W. M. Herriott—Don't use it so fast as to produce the heat.

Dr. Siddall—It don't cut much then.

Dr. Herriott—Use sharper burrs.

Dr. J. H. Warner had used the Morrison engine for three months, but found no way to obviate the heat except to use it more slowly, as suggested by Dr. Herriott.

Dr. C. R. Taft said every one who had used the burr knows of course that if put on a large gold filling, and held there continously, it would produce considerable heat. He had been in the habit of holding it off the teeth perhaps half the time; in that way it had a chance to cool off. It might be objected that this was a waste of time; but the time lost will not amount to much; and by thus keeping it off the tooth one-half or one third of the time, the difficulty complained of would be entirely obviated.

Dr. W. M. Herriott wished to say in this connection to those who had not used the engine—as those who had used it knew it to be true—that they would by its use save a great deal of pain to the patient, because there was less power required than with a hand drill or burr, or excavator, and also less pain because so much more speedy in its operation.

Dr. B. F. Rosson would like to encourage all the members of the profession who had not already made use of the burring engine to do so. He endorsed all the gentlemen said with regard to its lessening the pain of the patient. Of course the heating must be obviated or that would produce pain. His experience for the past four or five months had proven to him, that so readily and quickly are the operations performed by this means that the patient does not become weary. All know that we can sit down under an operation and tolerate the pain for a few minutes, but when to complete that operation an hour or two hours are required the patient becomes weary. With the Morrison engine—and he did not wish to advertise it more than others —but in his use of it he knew he could greatly lessen the pain to patients.

Dr. I. Williams found it admirably adapted to open up pulp cavity.

Dr. Gray said for polishing it was perfectly charming; also for cutting solid dentine. He had used it for five months, and could not consent to do without it.

Dr. D. W. Clancy found great difficulty in getting the burrs to run true. He always adjusted the rubber dam before commencing an operation.

---

THE QUIZ, Wednesday Evening, Dec. 4, 1872—Conducted by Dr. W. M. Herriott.

Dr. Herriott in a few introductory remarks announced that the quiz for the half hour allotted to it would be conducted with reference to the first topic discussed in the afternoon—The Burring Engine.

Dr. ———It is claimed, I believe that withthe burring engine sensitive dentine can be excavated without pain. I would like to know why.

Dr. Herriott—In talking upon the matter this afternoon it was said that sensitive dentine could be excavated with it with less pain, not without pain. It is, I suppose, on this principle, that if a tooth be hanging to the gum, if it be pulled slowly, it will be more painful than if removed at once; or if we undertake to puncture the flesh or interfere with the nerves of the flesh, anything that will do it instantly or rapidly will produce less pain than if the operation be slower. It is by the quick motion of the burring engine, as I understand it, that the pain is lessened in excavating dentine. As remarked this afternoon, the burr drills are simply a succession of chisels in a revolving position, and the cutting by them is done so rapidly as to produce less pain than by the slower motion of the hand operator.

Dr. B. F. Rosson thought perhaps the lessening of the pain was due to the fact that with the use of this instrument, less surface was run over at the same time.

Dr. Herriott admitted that this might be another cause of the lessening of the pain, and also because there was less pressure necessary with the use of this instrument than with the hand drill.

Question by a member—What is the best appliance to be used to polish a filling aiter the burring engine has been used?

Dr. Buffett—I should use a little felt wheel or a wheel made of leather, or a wash made of pummice, and finish it up with a little whiting, or with rouge, if I wanted to make a very nice finish.

A member—I have used leather, but found that as soon as it gets wet it softens down out of shape.

Dr. Buffett—Take the very hardest kind of sole leather.

He remarked in regard to burnishing, that you can not burnish anything with great pressure. You can scratch it, but can not burnish it.

Dr. Herriott—Dr. Harroun what difficulties do you find in the use of the burring engine as an excavating machine?

Dr. Harroun—That is a pretty broad question, to take it all around. The main difficulty I find is the heating of the tooth by the motion of the instrument—at least this is the main trouble my patients complain of—the only sensations they repudiate. The only difficulty I have had in making excavations is breaking the force of the instrument. I never had any success with what is called the back action, from the fact that I did not have any little burrs sent me with mine. I find some difficulty in getting it into some portions of the mouth especially in the lower jaw. I do not know that I find any particular difficulty in the general shape of the excavation. I don't depend upon the burring engine for the final shaping of the cavity. I use the burr only in cutting down portions of the enamel in order to make the walls more firm and solid to pack against.

Dr. Herriott remarked that in the discussions on the subject, at which Dr. Harroun was not present, it was suggested that the heat referred to could be obviated by using the engine more slowly, or by frequently ceasing its motion.

Dr. Harroun—I obviate it in another way. I use a spring that works easily, keeping it in the left hand, letting a drop of water fall upon it occasionally. I can work that way rapidly without pain to the patient.

Dr. C. R. Taft suggested that in the use of the burring engine, it wanted to be run rapidly, so as to cut quickly; but the operator should not bear on with great force. It would not cut as rapidly when thus bore on heavily, as if it ran rapidly and touched lightly.

Dr. Harroun—I noticed where there was some very sensitive teeth, so that you could hardly touch them with the instrument without making the patient scream, that this sensitiveness was almost entirely gone after I began to use the instrument. I would like to know whether the heat produced by the use of the instrument obtunded the sensitiveness. I had an idea it did.

The conductor of the Quiz requested Dr. L. Buffett to give his opinion in regard to this question.

Dr. Buffett—I would answer that you would not obtund it by any heat you would bring upon the teeth. If the burr is run rapidly and touched lightly, the heat thus produced I don't think would cause any pain.

Question—Would it allay the pain?

Dr. Buffett—The more rapidly you can run the engine, and keep the temperature low, the less pain there will be. If however it is run rapidly and produces much heat, it will give pain of course.

Dr. Rosson remarked that Dr. Blount, of Springfield, stated before the Miami Valley Association, that by the use of carbolic acid, an abscess might be opened without pain. Dr. Rosson said he had no experience in the matter himself, but thought it worthy of being mentioned here.

The time for this exercise having expired, the discussion on the second topic was resumed.

(To Be Continued.)

